# Environmentally Specific Servant Leadership and Employees’ Energy-Specific Pro-Environmental Behavior: Evidence from Healthcare Sector of a Developing Economy

**DOI:** 10.3390/ijerph19137641

**Published:** 2022-06-22

**Authors:** Junjie Peng, Sarminah Samad, Ubaldo Comite, Naveed Ahmad, Heesup Han, Antonio Ariza-Montes, Alejandro Vega-Muñoz

**Affiliations:** 1 Conservatory of Music, Guangxi Normal University, Guilin 541000, China; pjj15903750105@163.com; 2 Department of Business Administration, College of Business and Administration, Princess Nourah Bint Abdulrahman University, Riyadh 11671, Saudi Arabia; sarminasamad@gmail.com; 3 Department of Business Sciences, University Giustino Fortunato, 82100 Benevento, Italy; u.comite@unifortunato.eu; 4 Faculty of Management, Department of Management Sciences, Virtual University of Pakistan, Lahore 54000, Pakistan; naveed.ahmad@vu.edu.pk or; 5 Faculty of Management Studies, University of Central Punjab, Lahore 54000, Pakistan; 6 College of Hospitality and Tourism Management, Sejong University, 98 Gunja-Dong, Gwanjin-Gu, Seoul 143-747, Korea; 7 Social Matters Research Group, Universidad Loyola Andalucía, C/Escritor Castilla Aguayo, 4, 14004 Córdoba, Spain; ariza@uloyola.es; 8 Public Policy Observatory, Universidad Autónoma de Chile, Santiago 7500912, Chile; alejandro.vega@uautonoma.cl

**Keywords:** leadership, healthcare, sustainability, de-carbonization, climate change

## Abstract

Environmental issues are significantly rising worldwide. Addressing the environmental issues and preserving the biosphere is a critical matter of concern in this era. The sheer amount of total greenhouse gas (GHG) emissions in the world is related to the energy sector, especially electrical energy. A bulk of electrical energy is consumed by individuals in buildings for cooling and heating purposes. Prior researchers have emphasized employing clean and green energy sources to deal with environmental issues. The role of green energy from a decarbonization aspect is unchallengeable. However, a critical gap in most energy-related studies exists in the available literature. That is, most of the literature focuses on the supply side (the production) of energy, neglecting the critical issue lies with the demand side (consumption side). Energy data show that a sheer amount of electrical energy is wasted by individuals due to their inadequate energy consumption behavior. In this respect, a country’s healthcare system uses a significant amount of electrical energy. In particular, hospital staff uses a bulk of electricity during patient treatment, care, and other service delivery operations. The critical aim of this study is to improve the energy-specific pro-environmental behavior (EPEB) of hospital employees in an environmentally specific servant leadership (ESL) framework. Specifically, the study was conducted in Pakistan, which is a developing country. This study also tests the mediating effect of green self-efficacy (GSE) and green perceived organizational support (GPOS) in the above-proposed relationship. The data for the current work were collected from hospital employees by employing a survey strategy (*n* = 316) from a developing country. Structural equation modeling was considered to analyze the data, which confirmed that a servant leader with environmental preferences could significantly drive the EPEB of employees (*β* = 0.699), while GSE (*β* = 0.138) and GPOS (*β* = 0.102) mediated this relationship. The findings of this study can help the healthcare sector to improve its efforts toward de-carbonization by improving the energy consumption behavior of employees through ESL, GSE, and GPOS.

## 1. Introduction

Vulnerable climatic conditions that prevail in most societies and regions of the world have become a major challenge. The matter is especially problematic in several developing and poor countries, as such countries do not have sufficient resources to combat climate change [1]. In a recent meeting, a UN delegation from different countries mentioned that the unprecedented climatic conditions pose a disproportionate burden on developing economies [2]. Rising temperatures, poor air quality, pollution, contaminated water, and several other issues have put the health of the masses in danger [3]. Preserving nature and the biosphere by improving carbon emissions is a critical challenge faced by Global South nations. In this vein, climate data indicate that efforts at every level are required to reduce CO_2_ emissions [4]. The energy sector worldwide is held responsible for increasing the world’s greenhouse gas (GHG) emissions. A UN report estimated that around 35% of the world’s GHG emissions are associated with the energy sector. Perhaps this is one of the reasons why concepts such as clean and green energy have recently joined the academic lexicon. Multiple studies are available on clean and green energy, suggesting it as an alternative to reduce the CO_2_ emissions associated with the energy sector [5,6,7]. Considering the environmental severity associated with the energy sector, we feel that such studies are important to advance the debate on environmental management. Nevertheless, a critical gap exists in most energy-related studies. That is, the bulk of the literature deals with the supply side (production), neglecting the critical issue on the demand side (consumption). In simple words, the literature informs us that producing clean and green energy can reduce the environmental footprint of a country or region. However, the debate on how the efficient use of energy at the level of individuals can help a sector, region, or economy to deal with environmental issues is missing from the literature.

We feel that such a debate is important, at least for two reasons. First, the environmental data suggest that 60% of the total GHG emissions in the world are associated with the consumption pattern at an individual level [8]. This implies that the role of the individuals is critical in reducing the overall environmental dilapidation. Second, from an energy consumption perspective, most of the energy is consumed by individuals for heating and cooling purposes in a building. To make the matter more severe, it is estimated that, if not managed efficiently, the emissions from heating and cooling devices may rise by 90% in 2050 [9]. This again shows the critical importance of individuals from an environmental perspective.

Promoting sustainable individual behavior, which is also known as pro-environmental behavior (PEB), is a topic of hot academic debate in modern times [10,11,12]. Nonetheless, most of the literature has approached PEB from a general perspective (for example, improving the PEB of individuals through recycling, waste management, resource conservation, etc.). Considering the environmental issues related to the energy sector and considering the literature gap on the demand side, it is important to advance the debate on PEB by shifting from a general to an energy-specific approach. Hence, the critical objective of this research is to investigate the factors that can improve the energy-specific PEB (EPEB) of employees (individuals) in an organizational context.

ESL human behavior is complex to understand and is influenced by different factors [13,14]. In an organizational milieu, different organizational and personal factors influence the behavior of an individual. For example, the literature indicates that organizational factors such as corporate social responsibility engagement of an organization [15,16], organizational culture [17], organizational support [18], and leadership style [19] can significantly influence individual behavior. Similarly, the role of personal factors such as values [20,21], personality [22], emotions [23], and motivation [24] was also highlighted in the previous literature. Recently, organizational interest in understanding the role of corporate leaders in influencing employee behavior has increased [25,26]. In particular, the role of an environmentally specific servant leadership style (ESL) to spur the PEB of employees was realized at different levels [27,28]. We extend this debate by investigating if an environmentally specific servant leader can drive the EPEB of employees in an organization. Similarly, the mediating role of self-efficacy of employees (a personal factor) was also highlighted by previous behavioral scientists [29,30]. Nevertheless, green self-efficacy’s (GSE) mediating role in influencing PEB, especially EPEB, was not tested earlier in a leadership framework. Our intention was to fill this knowledge gap by exploring the mediating role of GSE between ESL and EPEB.

Likewise, another psychological factor that shapes employees’ behavior is known as perceived organizational support (POS). The mediating role of POS in influencing different employees’ outcomes was mentioned by prior researchers [31,32]. Even from an environmental perspective, the debate on green POS (GPOS) also exists [33,34]. Nevertheless, the important mediating role of GPOS in a leadership framework to spur the EPEB of employees was previously ignored. Therefore, this study introduces GPOS as a potential mediator between ESL and EPEB.

## 2. Literature Review and Development of Hypotheses

This study uses the theoretical lens of the theory of social learning to propose different hypothesized relationships. The theory of social learning was originally introduced by Bandura and colleagues [35], who stated that the social behaviors on the part of individuals take shape by observing the behaviors of others. From an organizational perspective, the milieu in which a corporate leader interacts with employees influences their behavior. The early behavioral theorists extensively used the theory of social learning to explain individual behavior in a certain context [36,37,38]. Even in a leadership framework, many scholars found this theory helpful in explaining the leader–member (employee) relationship [39,40]. Specifically, the scholars employing the theory of social learning argued that a corporate leader influences the behavior of employees through a process of social learning [41]. Extending this debate on the leader–member relationship from the theory of social learning perspective in the current context, we feel that a servant leader with an environmental preference conveys it to the employees through their actions to preserve the environment and biosphere. Employees as social observers, when seeing this environmental orientation of their servant leader, are motivated to learn this care for the environment from their leader. Thus, they are also expected to engage in acts which produce minimum or no harm to the environment.

Generally, a servant leader is one who puts forth the employees’ needs in the first place, rather than considering their own needs. Specifically, a servant leader lives with the premise that a leader needs to prioritize the employees’ interests over self-interest [42]. With a special focus on morality and ethics, a servant leader represents an ethical leadership perspective in which service is deep-rooted in leader–employee relations [43]. Frick [44] believed that, enriched by ethics, morality, and empathy, a servant leader is well placed to influence the behavior of employees in an organization. Sousa and van Dierendonck [45] presented servant leadership as action-driven with a special focus on the moral virtue of humility. From an environmental perspective, ESL can be regarded as a leadership style in which a corporate leader prefers environmental concerns over economic benefits by focusing on and promoting environmental values among employees [46]. The literature has generally established a positive link between servant leadership style and different employees’ discretionary outcomes (PEB is also a discretionary behavior) [47,48]. Some researchers even documented a positive relationship between servant leadership style and PEB [49,50]. Hence, we expect that a servant leader with an environmental orientation, especially for energy consumption, can drive the EPEB of employees in an organization.

**H1.**  *There exists a positive association between environment specific servant leadership and employees’ energy-related pro-environmental behavior.*

As one of the psychological factors, the literature indicates that self-efficacy can influence individual behavior in an organizational context [51]. Defined by Bandura [52], self-efficacy is the belief of an individual in their abilities to successfully complete a specific task. From an environmental context, GSE can be referred to as “the belief of an individual in their abilities to complete different tasks in an environmentally friendly manner”. Specifically, the literature mentions that self-efficacy from an environmental perspective can drive the PEB of employees in an organization [27,53]. Huang [54] believed that the self-efficacy of employees toward the environment could influence all kinds of PEB of employees. Kim, et al. [55] went to great lengths to establish a positive association between GSE and the PEB of employees. Kim and colleagues also mentioned that, in an organization, employees with greater GSE perceptions hold a strong belief that their specific environmental actions could support their organization in efficiently dealing with the environmental issues. The same kind of assertion can be seen in the work of Tabernero and Hernández [56]. Other scholars have also established a positive link between GSE and employees’ PEB [28,57].

The literature also suggests that different organizational factors influence the self-efficacy of employees. Among such organizational factors, scholars have gone to great lengths to document the positive impact of leadership style on employees’ self-efficacy [58,59]. Especially from an environmental perspective, ESL was associated with the GSE of employees at different levels [60,61]. The mediating role of GSE to spur employees’ PEB also exists in the available leadership literature [62,63]. In this respect, we feel that a servant leader with environmental preference can influence employees’ PEB, especially their EPEB. A servant leader shows the employees that they prefer environmental benefits over economic efficiency. Furthermore, enriched with a service-like attitude, a servant leader helps their followers to achieve different organizational objectives in an environmentally friendly manner. Referring to the process of social learning, the environmental preference of a servant leader is expected to be imitated by the workers. This process guides them to show great concern for the environment, which motivates them to act pro-environmentally. More specifically, a servant leader with environmental preferences takes different steps toward efficient energy consumption in a workplace. In this respect, the same social learning process can be referred to as inculcating an energy-friendly behavior of employees. The above discussion can be summarized by the following hypotheses:

**H2.**  *There exists a positive relationship between green self-efficacy and employees’ energy-related pro-environmental behavior.*

**H3.**  *Green self-efficacy is expected to mediate the relationship between environment-specific servant leadership and employees’ energy-related pro-environmental behavior.*

A corporate leader with a high environmental orientation inculcates this feeling among employees that their organization provides a supportive environment in which employees’ environmental contributions can be evaluated positively [64]. Employees’ perception that an organization supports green initiatives is critical for motivating them to act in an environmentally friendly manner [65]. Furthermore, GPOS perceptions of employees urge them to facilitate their employer in achieving different environmental objectives. Shabbir, et al. [66] believed that the GPOS of an organization helps its employees in developing positive feelings to think that their environmentally friendly act will be rewarded by their employer. Shoss, et al. [67] regarded POS as a critical factor that motivates employees to show a great commitment to overall organizational success. Kurtessis, et al. [68] mentioned that POS not only fulfills employees’ socioemotional needs but also leads them to show greater environmental commitment to their organization by acting pro-environmentally. The presence of a servant leader in an organization further improves employees’ POS perceptions [69]. Indeed, from an environmental aspect, Luu [70] confirmed a significant indirect effect of GPOS on influencing employees’ discretionary behavior in an ESL framework. Huning, et al. [71] also confirmed that POS mediates between servant leadership style and employees’ outcomes. Other scholars have also documented a significant indirect role of POS in influencing the servant leader–member relationship [69,72]. Advancing this debate from an environmental aspect, we expect that a servant leader with environmental values not only directly influences employees’ EPEB but also improves employees’ feelings that the organization supports their green initiatives. They also effectively communicate to the employees that the organization provides a supportive green environment, and that, if they support the organization in achieving its environmental objectives, they will be positively evaluated. In a nutshell, we can propose the following hypotheses:

**H4.**  *There exists a positive relationship between green perceived organizational support and employees’ energy-related pro-environmental behavior.*

**H5.**  *Green perceived organizational support is expected to mediate the relationship between environment-specific servant leadership and employees’ energy-related pro-environmental behavior.*

**Figure 1:**
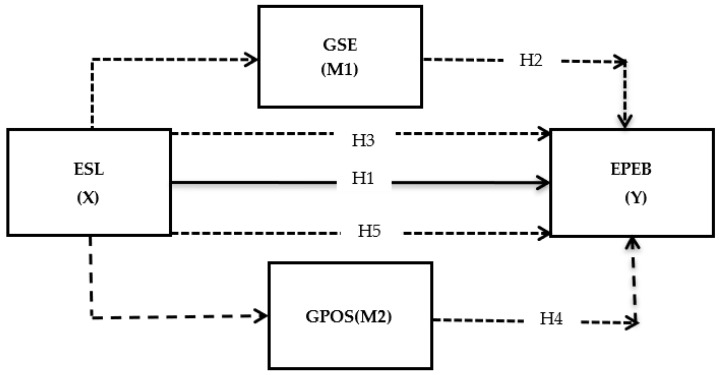
Theoretical framework showing different relationships. The dotted lines show the direct relationships in the presence of mediators. The bold lines show the total effect between predictor and criterion. ESL = environment-specific servant leadership, EPEB = energy-specific pro-environmental behavior, GSE = green self-efficacy, GPOS = green perceived organizational support.

The hypothesized relationships are presented in Figure 1. 

## 3. Methods

### 3.1. Participants and Procedure

The target focus of this study was the healthcare sector of Pakistan. As a developing country, this South Asian nation is currently facing severe climatic conditions [64,73]. The current environmental severity in the country requires active participation from all segments, with no exceptions for the healthcare system. Indeed, the healthcare system is directly or indirectly associated with pollution [74]. Considering the 24/7 service delivery nature of hospitals, the energy requirement in this sector is huge. During patient treatment, care, and other services, the hospital staff consumes a large amount of electricity. The hospital sector in the country includes both public and private hospitals. These hospitals operate in the country under four different mechanisms: curative, preventive, rehabilitative, and promotive health services. It was estimated that around 80% of patients are attended by private hospitals [75].

Considering the labor intensity in this sector and realizing its outsized energy consumption, it is important to improve the EPEB of employees in this sector. According to an estimate, several hundred thousand employees work in the hospitals of Pakistan [76]. We identified Lahore and Karachi as sampled cities to collect the data. Both cities are provincial capitals and constitute a population of several millions people. At the same time, both of these cities hold the largest umbrella of public and private hospitals. However, the population and large number of hospitals were not the only reasons for identifying these cities as a base. The critical point that makes these two cities most relevant for this survey was the poor environmental conditions in these two cities. Indeed, Lahore and Karachi are included in the list of most polluted cities worldwide. In particular, Lahore is known as the world’s most polluted city [77].

To proceed with data collection, we contacted the management of different hospitals to facilitate this survey. Some hospitals responded positively, while others did not. Those who responded positively were then approached by us to advance the data collection. A total of eight hospitals were included in this survey. Specifically, the data were collected between September and November 2021.

### 3.2. Measures

The variables in this study (ESL, EPEB, GSE, and GPOS) were measured by adapting items from different sources. Given that their reliability and validity are known, we followed other authors in using pre-existing scales [26,78].

We employed the definition by Tuan [79] to define ESL as “a corporate leader who keeps environmental interests ahead of personal and organizational interests (especially economic interests) by promoting pro-environmental values among organizational stakeholders”. This variable was measured using 12 items from Liden, et al. [80]. The items of this scale measured the perceptions of employees about their supervisor/manager with respect to environmental management. Sample items from this scale were “My supervisor/manager encourages me to contribute eco-initiatives” and “My supervisor/manager always displays green behaviors”. The inter-item consistency of this scale was maintained by observing the α value, which was 0.927.

We conceptualized EPEB by following the definition of Lopes, et al. [81] who defined it as “a set of individual actions that influence energy consumption and production”. To measure EPEB, we adapted eight energy-related items from Blok, et al. [82]. Examples included “This employee ensures that heating/air conditioning is off or reduced outside working hours” and “This employee switches off their computer/notebook when they leave the office for a considerable period”. The α value for this scale was 0.889, which was significant.

GSE is the belief of an individual in their abilities to complete different tasks in an environmentally friendly manner. We measured this variable using the scale of Chen, et al. [83], which consisted of six items. The scale measured the extent to which employees perceived they were able to help an organization in achieving its environmental goals and mission. Sample items from this survey included “I can achieve most environmental goals” and “I can perform effectively on environmental missions”. A significant α value = 0.872 was observed in this case.

Following the definition by Eisenberger, et al. [84], we defined GRPS as “the perception of an employee that the organization values their environmental contribution and takes care of their wellbeing”. A four-item scale was adapted from Eisenberger, et al. [84] to measure GRPS with α = 0.842. One sample item was “This hospital really cares about my environmental goals and values”. The data were collected on a five-point Likert scale. Appendix A includes the list of all survey items.

### 3.3. Instrument

An adapted questionnaire (self-administered) was used in this survey to collect the data from different respondents who were hospital employees and managers/supervisors. The adapted questionnaire items were assessed by field experts (academia and hospitals), as recommended by previous scholars [85,86]. The layout of the questionnaire was twofold. In the first part, the sociodemographic information of the respondents was obtained, whereas the survey item-related information was obtained in the second part. To maintain the ethicality in this survey, we followed the Helsinki Declaration’s ethical guidelines, as also asserted by previous scholars [87,88].

A three-wave data collection strategy was followed to collect the information from the respondents. Each wave was administered with a 2 week interval. Specifically, sociodemographic information and GSE were evaluated in the first wave, whereas ESL was evaluated in the second wave. Lastly, EPEB and GPOS were evaluated. More specifically, employees rated their manager/leader, whereas managers were invited to comment on the EPEB of an employee under their supervision. We initially distributed 600 surveys; however, we received close to 53% valid responses (*n* = 316). The sociodemographic information is presented in Table 1.

## 4. Results

### 4.1. Reliability and Validity of the Variables

The successful completion of the data collection stage led us to proceed with the data analysis phase of this study. We, first of all, evaluated the studied variables in terms of reliability and validity. In this respect, we executed a confirmatory factor analysis (CFA) in AMOS software. This was performed to confirm whether the factor loading of each item was significant or not (significant for λ > 0.7). The outcomes of CFA confirmed that the factor loadings of all items were significant. For example, the variable GPOS constituted four items with loadings of 0.833, 0.703, 0.724, and 0.838. These loadings were positive and significant, confirming that there was no issue in the factor loading of any item belonging to GPOS. The overall factor loadings of all variables varied from 0.700 to 0.838. As all factor loadings were significant, we did not drop any item of a variable. The significant results of CFA provided an initial base for the establishment of convergent validity (CoL) and composite reliability (CR), because these factor loadings were helpful in calculating the average variance extracted (AVE) value of a variable.

It was observed that all AVEs were positive and beyond the standard cutoff value of 0.5 (AVE = 0.586, 0.574, 0.589, and 0.604 for ESL, EPEB, GSE, and GPOS, respectively). The general practice to confirm the CoL for a variable is to observe its AVE. As all AVEs were significant, the CoL for all variables was confirmed. Similarly, the factor loadings during CFA were also used to calculate the CR value of a variable. It was observed that all CRs were significant (>0.7). Specifically, the CR values ranged from 0.944 to 0.858. 

### 4.2. Alternate vs. Hypothesized Models

Furthermore, we prepared three alternate measurement models to compare with the baseline model. This step was taken to see whether any of the alternate models produced superior model fit values in comparison to our baseline model. In this respect, it was realized that the hypothesized four-factor model was superior in terms of model fit indices and other model fit values (NFI = 0.928, CFI = 0.930, *χ*^2^*/df* = 2.581, and RMSEA = 0.062).

### 4.3. Correlations

Measuring correlation values between different pairs of variables was the next stage in data analysis. The output of correlation analysis using AMOS revealed that correlations (*r*) were positive and significant. Specifically, the *r*-values ranged from 0.283 (GSE→GPOS) to 0.496 (ESL→GPOS). Furthermore, the *r*-values were moderate (<0.7), indicating that the dataset of this study did not suffer from the critical issue of multicollinearity. Furthermore, we also examined the discriminant validity (DvL) for each variable, which ranged between 0.765 (ESL) and 0.777 (GSE). All of these values were significant, establishing that the items of one variable did not match with other variables. We refer to Table 2 for more detail on correlation values and DvL.

### 4.4. Total, Direct, and Indirect Effects

For hypothesis analysis, we used structural equation modeling (SEM). Specifically, we developed two structural models: one which did not include any mediator and the other with mediators (Table 3). In this respect, a direct effect structural model was evaluated in the first place. This model showed significant beta values (*β*). We were able to validate H1, H2, and H4 of this study through this direct effect model (*β* = 0.699, 0.328, and 0.349 for H1, H2, and H4; *p* < 0.05 in all cases). Similarly, nonzero confidence intervals (lower limit and upper limit) in each case were also a confirmation that H1, H2, and H4 were statistically significant.

The mediating effects of GSE and GPOS were tested in the second phase of structural analysis using a large bootstrapping sample [89,90,91]. The results of this structural model showed that GSE and GPOS significantly mediated the relationship between ESL and EPEB (*β* = 0.138, *z* = 08.625, *p* = 0.000, CI = 0.115–0.172; *β* = 0.102, *z* = 10.200, *p* = 0.007, CI = 0.091–0.180, respectively). These values confirmed that H3 and H5 were also significant.

## 5. Discussion

Considering climatic vulnerability in Pakistan and considering the role of individuals in preserving the nature and biosphere, this study advances the debate on PEB from an energy-specific context. As with other nations of the Global South, Pakistan is ranked lower in the list of nations with improved sustainability conditions. The country needs support from all sectors of the economy in its sustainability initiatives for a better and sustainable future. The results showed that ESL can influence the EPEB of employees in an organization. Specifically, the healthcare staff uses a bulk of energy during different health delivery services. This energy consumption behavior of healthcare employees related to this sector can be improved through different organizational factors, including leadership style and a supportive green environment.

The statistical result of our study showed that a servant leader with high environmental values could positively influence the EPEB of employees (*β* = 0.699). With a special focus on morality and ethics, a servant leader represents an ethical leadership perspective in which service is deep-rooted in leader–employee relations. Specifically, ESL can be viewed as a person who prefers environmental benefits over economic benefits. At the same time, they show their environmental commitment through energy consumption behavior. Following the social learning process, employees learn such energy-efficient behavior from their leader, which ultimately urges them to practice this behavior. This process improves their EPEB.

Another aspect of this study was to highlight the role of GSE as a mediator between ESL and EPEB. In this respect, the statistical results showed that GSE not only directly influences EPEB (*β* = 0.328), but significantly mediates the relationship between ESL and EPEB (*β* = 0.138). Specifically, this mediation effect accounts for a 20% variation in EPEB. In this respect, on one hand, a servant leader helps employees to improve their energy consumption behavior by guiding and facilitating them. On the other hand, employees’ belief that their energy consumption behavior can meaningfully support their organization also influence their EPEB. Lastly, our study also confirmed the mediating effect of GPOS for the relationship between ESL and EPEB. As a mediator, GPOS accounted for 14.59% variation in EPEB (*β* = 0.102). A servant leader infuses this feeling among employees that their organization provides a supportive green environment in which employees’ environmental contributions will be evaluated positively. Employees’ perception that an organization supports green initiatives motivates them to act environmentally friendly, especially by reducing their energy consumption level. At the same time, GPOS perceptions of employees urge them to facilitate their organization in achieving different environmental objectives. The results confirmed that, from an energy perspective, a servant leader not only influences employees’ EPEB directly but also improves employees’ feelings that the organization supports their green initiatives. Ultimately, employees working under the supervision of an ESL with improved GPOS perceptions are motivated to show greater commitment to an enhanced level of EPEB.

### 5.1. Implications

Considering the environmental severity associated with the energy sector, our study attempts to fill the critical knowledge gap on the energy demand side (consumption side). In this vein, as stated earlier in the onset of this document, the bulk of the literature deals with the supply side of energy (production-side). Specifically, our study advances the theoretical framework of Mughal, et al. [27], who highlighted the energy crisis in Pakistan and mentioned the role of a servant leader to spur the general PEB of employees. Although their study considered the mediating effect of GSE, they did not approach the PEB of employees from an energy-specific standpoint. Similarly, another important contribution of our study is to advance the debate on environmental management in the healthcare context of Pakistan (a Global South nation). Considering the labor intensity in this sector and realizing its outsized energy consumption, it is important to improve the EPEB of employees in this sector. However, in prior studies, the researchers did not approach the healthcare system of Pakistan from an energy efficiency perspective. Although scholars reported the overuse of energy in this sector [92,93], they also subscribed to the supply side view of energy, neglecting the issue on the demand side. Lastly, considering the complex nature of human behavior, our study is among the sparse studies that considered both organizational and personal factors to explain the PEB of employees from an energy efficiency perspective. More specifically, to the best of our knowledge, this is the first study to advance the available literature by considering the simultaneous mediating effects of GSE and GPOS in a unified servant leadership model to explain the EPEB of employees.

On a practical landscape, our research helps the healthcare sector of Pakistan to improve its environmental footprint by promoting sustainable energy consumption behavior among employees through ESL. Considering the sheer amount of GHG emissions associated with individuals and considering the likelihood that, if not managed, the emissions from air-conditioning and heating devices will increase by 90%, it is a matter of concern to improve the energy consumption and preservation behavior of every individual, including employees. Moreover, the healthcare sector is one of the service sectors known as a large energy consumer. The 24/7 service delivery nature, patient care and treatment, and labor intensity of this sector are some significant reasons why this sector is identified as a large user of energy, especially electrical energy. In this respect, the healthcare staff has a clear role in helping a hospital in improving its steps toward de-carbonization. From this standpoint, we suggest that the management of a hospital carefully promotes the essence of a servant leadership style among employees with managerial ranks. More specifically, the hospital management needs to emphasize the energy-saving behavior within its leadership because, when characterized by a service-rooted nature, a servant leader is central to influencing employees’ behavior, including EPEB.

On a further note, it is also important for a hospital administration to improve the GSE perceptions of employees because employees with a higher level of GSE will be passionate about contributing more to different environmental causes, including energy consumption. To do so, we suggest that a hospital organization may arrange special training programs to improve the GSE perceptions of employees under the framework of ESL.

Similarly, hospital administration requires infusing this notion among employees that a certain hospital is very supportive of providing a green environment. To do this, we urge hospital management to provide a greener workplace environment (especially by installing energy-saving electrical equipment) so that the workers get this clear message that they are not only keen to provide a greener environment but also supportive of the employees in achieving a sustainability initiative. To sum up, specific hospitals need to improve their environmental efficiency, for which the role of employees is seminal. In this respect, an effective leadership style (for example, ESL) and different organizational (for example, GPOS) and personal (for example, GSE) traits are critical to improving the energy-specific sustainable behavior of employees.

### 5.2. Limitations and Future Research Guidelines

There are a few potential limitations of this study that we want to highlight. First of all, due to a lack of access to any sampling frame, we employed a nonprobability sampling method (convenient sampling), which is considered inferior to probability sampling. In this respect, we suggest that future studies adopt a probability sampling method. Second, this study only collected data from two large cities in Pakistan; it is suggested that a larger geographical area be covered by including more cities in future studies. The cross-sectional data collection strategy is also an issue that serves as a potential limitation. In this respect, we suggest following a longitudinal data collection strategy in future studies. Furthermore, this study was conducted only in Pakistan; however, other countries in the same region, for example, India and Bangladesh, also face the same environmental and energy crises currently faced by Pakistan. Interestingly, these countries also have many things in common, such as culture, education levels, systems, and population characteristics. Therefore, it will be worthwhile to produce a comparative analysis (if possible) of India, Pakistan, and Bangladesh. Lastly, the transition of different variables from general to specific, e.g., self-efficacy, could impact the association.

## 6. Conclusions

Being included in the list of Global South nations, the environmental conditions in Pakistan are not good. Indeed, the country is one of the nations facing the worst climatic conditions. Moreover, the energy crises in Pakistan are also huge. The sheer production of electricity using fossil fuels is a critical point contributing to the increase in poor environmental conditions. Furthermore, the inefficient energy consumption behavior at the level of individuals puts pressure on electricity-producing companies, contributing to more environmental hazards. For a better and more sustainable future, Pakistan requires all sectors of the economy to show a responsible attitude toward achieving sustainability initiatives. From the perspective of Asia, especially the South Asian region, this study advances the debate on environmental management and the energy crisis in India and Bangladesh. Indeed, these countries have many things in common (as all three countries were a single region in the past, known as a subcontinent) and, unfortunately, face the same poor environmental and energy crisis [94,95]. Moreover, according to a recent estimate [96], more than 25% of the world resides in South Asia. Hence, improving the energy-specific behavior of such a huge population in a leadership framework is worthwhile to improve the environmental footprint of this region. Studies from other regions in the world have also identified the role of an effective leadership style (such as ESL) to improve the pro-social behavior of employees [70]. The important takeaway of this study is to highlight the critical issue with respect to the demand side of energy consumption, for which the attitude of individuals is vital. In a healthcare context, employees require the same caring attitude. To conclude, green and clean energy sources undoubtedly have a clear role in improving the environment and preserving the biosphere. However, the consumption pattern of individuals also requires attention.

## Figures and Tables

**Table 1:** Demographic profile of the sample.

Demographic	Frequency (*n* = 511)	%
**Gender**		
Male	199	62.97
Female	117	37.03
**Age**		
22–25	62	19.62
26–30	76	24.05
31–35	82	25.95
36–40	58	18.35
Above 40	38	12.03
**Experience**		
1–3	68	21.52
4–6	106	33.54
7–9	78	24.68
10 and above	64	20.25

Note: Age and experience were reported in terms of years.

**Table 2:** Correlations and discriminant validity.

Construct	ESL	EPEB	GSE	GPOS	Mean	SD
ESL	0.765	0.408	0.322	0.496	2.88	0.72
EPEB		0.757	0.419	0.386	3.02	0.64
GSE			0.777	0.283	2.92	0.70
GPOS				0.768	3.49	0.55

Notes: SD = standard deviation; diagonal = discriminant validity values; *p <* 0.005, 0.001.

**Table 3:** Total, direct, indirect, and conditional effects.

Hypotheses	Relationship	Estimates (SE)	*t/z*	*p*-Value	CI
Total effect (ESL→EPEB)	positive	0.669(0.054)	12.388	0.000	0.592–0.768
Direct effects					
(ESL→EPEB)	Positive	0.429 (0.4240)	10.117	0.005	0.399–0.533
(ESL→GSE)	Positive	0.421 (0.0428)	09.836	0.007	0.408–0.511
(GSE→EPEB)	Positive	0.328 (0.0392)	08.367	0.003	0.283–0.406
(ESL→GPOS)	Positive	0.293 (0.0220)	13.318	0.000	0.259–0.394
(GPOS→EPEB)	Positive	0.349 (0.0421)	08.309	0.000	0.303–0.386
Indirect effect					
(ESL→GSE→EPEB)	positive	0.138 (0.0160)	08.625	0.000	0.115–0.172
(ESL→GPOS→EPEB)	positive	0.102(0.0100)	10.200	0.007	0.091–0.180

Notes: CI = 95% confidence interval with lower and upper limits.

## Data Availability

The data will be made available on a reasonable request by contacting the corresponding author.

## Appendix A. Items Used in This Survey

**Items**
Environment-specific servant leadership
My supervisor/manager cares about my eco-initiatives
My supervisor/manager emphasizes the importance of contributing to environmental improvement
My supervisor/manager is involved in environmental activities
I am encouraged by my supervisor/manager to volunteer in environmental activities
My supervisor/manager has a thorough understanding of our company and its environmental goals
My supervisor/manager encourages me to contribute eco-initiatives
My supervisor/manager gives me the freedom to handle environmental problems in the way that I feel is best
My supervisor/manager does what they can to realize my eco-initiatives.
My supervisor/manager holds high environmental standards
My supervisor/manager always displays green behaviors
My supervisor/manager would not compromise environmental principles in order to achieve success
My supervisor/manager values environmental performance more than profits
Pro-environmenal behavior
This employee switches off the lights when they leave the office for a considerable period of time, and there is no one else
This employee switches off the heating or cooling devices when the outside climate is ok
This employee checks whether the thermostats of heating or cooling devices are set correctly in the office
Instead of using an air conditioner or a heating device, this employee wears suitable clothes to deal with the weather situation
This employee switches on the lights when they come to the office in the morning and switches them off before they leave
This employee switches off their computer/notebook when they leave the office for a considerable period
This employee switches off their computer/notebook when they go home
This employee makes sure that heating is off or reduced outside working hours
Green Self-Efficacy
I feel I can succeed in accomplishing environmental ideas
I can achieve most environmental goals
I feel competent in dealing effectively with environmental tasks
I can perform environmental missions effectively in
I can overcome environmental problems
I can identify creative solutions to environmental problems
Green Perceived Organizational Support
This hospital values my contribution to environmental management
This hospital really cares about my environmental goals and values
This hospital cares about my opinions on sustainability
This hospital takes pride in my accomplishments regarding environmental issues at work
